# The *In Vitro* Cytotoxic Effects of Ionophore Exposure on Selected Cytoskeletal Proteins of C2C12 Myoblasts

**DOI:** 10.3390/toxins14070447

**Published:** 2022-06-30

**Authors:** Danielle Henn, Annette Venter, Gezina C. H. Ferreira, Christo J. Botha

**Affiliations:** Department of Paraclinical Sciences, Faculty of Veterinary Science, University of Pretoria, Onderstepoort 0110, South Africa; annette.venter@up.ac.za (A.V.); arina.ferreira@up.ac.za (G.C.H.F.); christo.botha@up.ac.za (C.J.B.)

**Keywords:** C2C12 myoblasts, cytoskeleton, cytotoxicity, desmin, ionophores

## Abstract

Carboxylic ionophores, such as monensin, salinomycin and lasalocid, are polyether antibiotics used widely in production animals for the control of coccidiosis, as well as for the promotion of growth and feed efficiency. Although the benefits of using ionophores are undisputed, cases of ionophore toxicosis do occur, primarily targeting the cardiac and skeletal muscles of affected animals. The 3-[4,5-dimethylthiazol-2yl]-2,5-diphenyl tetrazolium bromide (MTT) viability assay was used to determine the cytotoxicity of monensin, salinomycin and lasalocid on mouse skeletal myoblasts (C2C12). Immunocytochemistry and immunofluorescent techniques were, in turn, performed to investigate the effects of the ionophores on the microfilament, microtubule and intermediate filament, i.e., desmin and synemin networks of the myoblasts. Monensin was the most cytotoxic of the three ionophores, followed by salinomycin and finally lasalocid. Monensin and salinomycin exposure resulted in the aggregation of desmin around the nuclei of affected myoblasts. The synemin, microtubule and microfilament networks were less affected; however, vesicles throughout the myoblast’s cytoplasm produced gaps within the microtubule and, to a limited extent, the synemin and microfilament networks. In conclusion, ionophore exposure disrupted desmin filaments, which could contribute to the myofibrillar degeneration and necrosis seen in the skeletal muscles of animals suffering from ionophore toxicosis.

## 1. Introduction

Carboxylic ionophores are polyether antibiotics widely used in the production animal industry due to their selective toxicity against bacteria and protozoan parasites, as well as their ability to improve feed efficiency in ruminants [1,2,3,4,5]. However, it is not uncommon for animals to ingest a dosage that exceeds safety margins, either resulting from feed-mixing errors or extra-label use, and subsequently suffer from ionophore toxicosis [6,7,8,9,10,11,12,13,14,15]. Treatment of ionophore toxicosis is largely symptomatic and supportive [12,13,14], and a poor prognosis is associated with suspected myocardial damage [11,16]. Ionophores act by forming dynamically reversible, lipid-soluble cation complexes and facilitating the movement of ions across biological membranes. The resulting changes in the net movement of ions within the cell disrupt ion homeostasis, causing, among others, calcium overload, production of reactive oxygen species (ROS) and disruption of cellular membranes [3]. Striated muscles are the primary targets of ionophore toxicosis, and clinical signs are thus mainly associated with skeletal muscle and myocardial dysfunction. Signs and symptoms of ionophore toxicosis include anorexia, ataxia, depression, diarrhea, dyspnea, hypoactivity, recumbency and weakness [6,8,12,16,17,18,19,20,21]. On auscultation of the heart, tachycardia and cardiac arrhythmias are present [6,8,11,15,17,18]. Lesions are time- and dosage-dependent and are often absent in animals that die immediately after ionophore exposure. Morphological effects of ionophore toxicosis on cardiac and skeletal muscle comprise degeneration, necrosis, and repair with a variable inflammatory component [7,8,21,22,23,24].

Ultrastructural examination of the skeletal muscles from sheep that suffered from experimental monensin toxicosis revealed disorganization of the sarcomere, exhibiting mild Z-disk streaming and indistinct A- and I-bands [22]. Further progression of ionophore toxicosis later developed into total disruption of the Z-disk with granular material visible. The sarcomere is the most basic contractile unit of a muscle, with actin (thin) and myosin (thick) filaments, working in tandem alongside various other proteins, adenosine triphosphate (ATP), and calcium to facilitate contraction. Desmin, a muscle-specific intermediate filament is found primarily at the Z-disk of the sarcomere and assists with maintaining muscle cytoarchitecture [25,26,27,28]. Additionally, desmin regulates the positioning of mitochondria within the cytoplasm, thus affecting their respiratory ability [29,30]. Synemin, another intermediate filament, co-polymerizes with desmin at the Z-disk and assists with structural support, connects desmin to the extracellular environment [31,32,33] and functions in various signaling pathways [34,35]. Although not part of the sarcomere, the microtubule network is associated with desmin at the Z-disk and mechanically couples the sarcomere to the non-sarcomeric cytoskeleton [36,37]. The aim of this study was to investigate the *in vitro* cytotoxicity of monensin, salinomycin and lasalocid on C2C12 myoblasts and to determine the effects of the ionophores on the cytoskeletal proteins, desmin, synemin, β-tubulin and actin.

## 2. Results

### 2.1. The In Vitro Cytotoxic Effect of Monensin, Salinomycin and Lasalocid on C2C12 Myoblasts

#### 2.1.1. The Effect of Ionophore Exposure on Cell Viability

**Figure 1:**
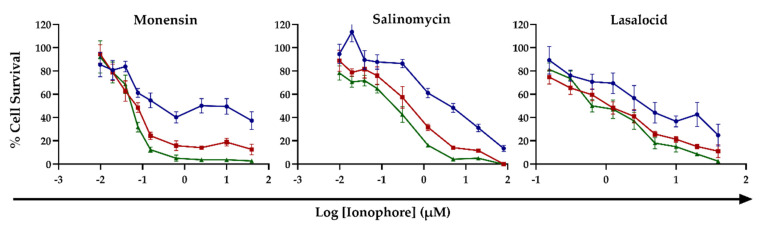
The log dose-response curves of C2C12 myoblasts exposed to monensin, salinomycin and lasalocid for 24, 48 and 72 h. Mean ± SEM. (Legend -●- 24 h, -■- 48 h, -▲- 72 h.).

The effect of monensin, salinomycin and lasalocid on C2C12 myoblast viability was investigated over a 72 h exposure period using the MTT viability assay. The ionophore toxicity was dose- and time-dependent, with the percentage cell survival inversely proportional to the ionophore concentrations and exposure duration (Figure 1). The log dose–response curves after 48 and 72 h exposure closely resembled each other, with the percentage cell survival being slightly lower after 72 h. Increased concentrations of both monensin and salinomycin resulted in a sharp decline in the percentage of viable cells after 48 and 72 h of exposure, whereas after 24 h exposure, ionophores exhibited a smaller effect on cell survival.

The half-maximal effective concentrations (EC_50_s) ranged from the low micromolar to low nanomolar range, decreasing sharply from 24 to 48 h exposure (Table 1). There were significant differences (*p* < 0.05) between the EC_50_s at 24 h ^a^ compared to the EC_50_s at both 48 ^b^ and 72 h ^b^ exposures of salinomycin and lasalocid, respectively. Of the three ionophores, monensin ^c^ was the most toxic, having significantly lower EC_50_s compared to both salinomycin ^d^ and lasalocid ^d^ after 48 and 72 h exposure (*p* < 0.05). Salinomycin was the second most cytotoxic ionophore tested, while lasalocid was the least cytotoxic, with EC_50_s remaining within the low micromolar range even after 72 h exposure. 

#### 2.1.2. Morphological Alterations of C2C12 Myoblasts

**Figure 2:**
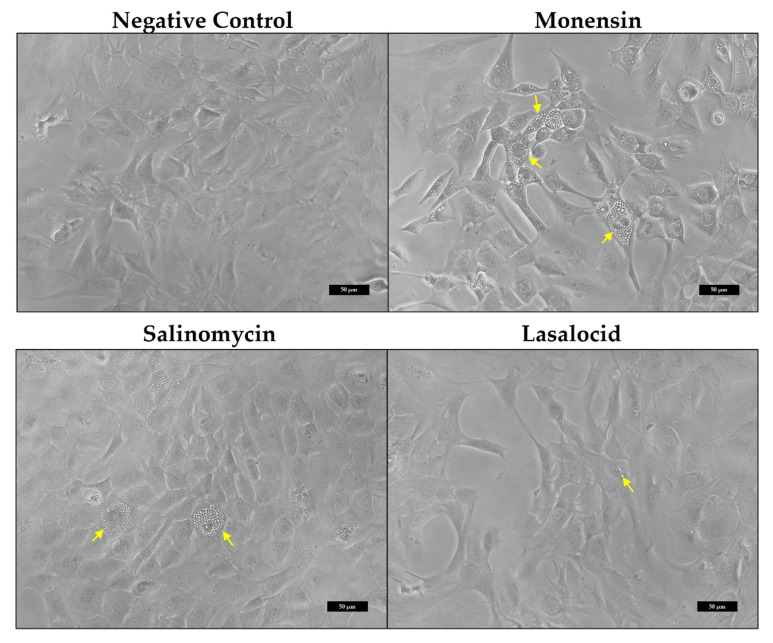
C2C12 myoblasts after exposure to 1 µM monensin, salinomycin and lasalocid for 48 h. The cytoplasm of exposed myoblasts becomes filled with translucent vesicles (yellow arrows). The scale bar = 50 µm.

Investigation of the myoblast morphology after ionophore exposure using phase-contrast microscopy (Figure 2) revealed that affected myoblasts were filled with translucent vesicles (yellow arrows). The vesicles initially appeared around the perinuclear region, eventually filling up the cytoplasm. Some myoblasts became spherical and detached from the surface. Monensin exhibited the greatest effect on the myoblasts (Figure 2), in terms of reduction in adherent myoblasts and with the majority of the myoblasts containing cytoplasmic vesicles. 

### 2.2. The In Vitro Effect of Ionophores on Desmin and Synemin in C2C12 Myoblasts

#### 2.2.1. Desmin

**Figure 3:**
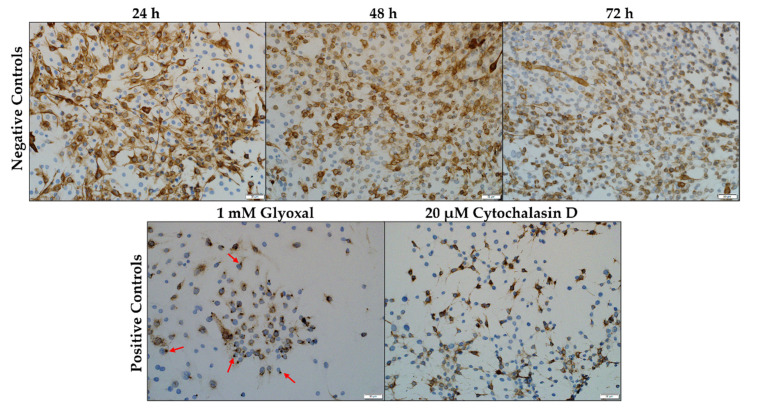
C2C12 myoblasts stained for desmin. The negative control consisted of C2C12 myoblasts incubated in DMEM medium over a 72 h period. Glyoxal and Cytochalasin D were used as positive controls. Desmin aggregates are indicated by the red arrows. Scale bar = 50 µm.

Desmin is seen in the C2C12 myoblasts, stained a light brown color following antibody binding and visualization with the chromogen, 3,3′-diaminobenzidine (DAB). In the negative control, desmin appeared as a diffusely filamentous network throughout the cytoplasm, exhibiting slight condensation in the perinuclear region (Figure 3). The increase in the number of myoblasts was also concomitant with an increase in incubation time. At 72 h, the labelling intensity of desmin seemed to have decreased slightly. The solvent control (0.1% methanol (MeOH)) (not shown) was similar to that of the negative control. Glyoxal (1 mM) and cytochalasin D (20 µM) were used as positive controls. Exposure to glyoxal caused a dot-like desmin aggregation pattern near the perinuclear region of the myoblasts (Figure 3, red arrows). In contrast, cytochalasin D exposure caused desmin to form filamentous protrusions radiating outwards from the nucleus.

**Figure 4:**
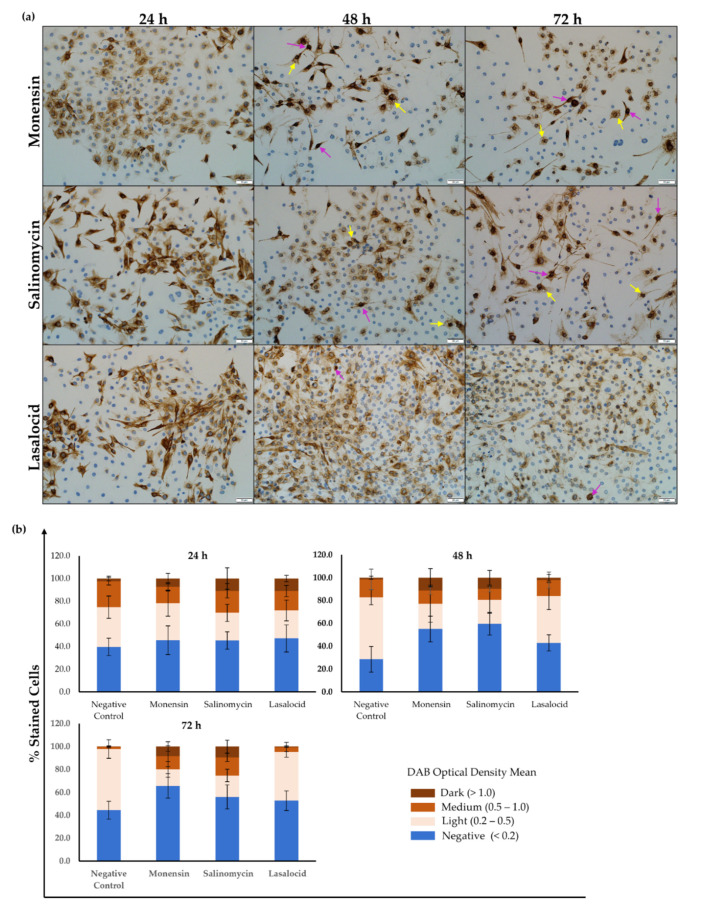
(**a**) C2C12 myoblasts stained for desmin after exposure to 1 µM monensin, salinomycin and lasalocid over a period of 72 h. Myoblasts that stained darker around the nucleus are indicated by purple arrows. Yellow arrows indicate vesicles/gaps within the desmin network. (**b**) Semiquantitative analysis of the desmin staining intensity.

Ionophore exposure affected the distribution of desmin within the C2C12 myoblasts (Figure 4a). After 24 h exposure, the myoblasts appeared similar to that of the negative control. Gaps (yellow arrows) were visible within the desmin network after 48 h exposure, particularly in myoblasts exposed to monensin and salinomycin. Desmin debris were also present, and the number of myoblasts on the slides was visibly less compared to that of the negative control. Myoblasts were stained darker around the nuclei (purple arrows), indicating possible perinuclear desmin aggregation, and appeared elongated compared to that of the negative control. Lasalocid exposure resulted in slightly fewer myoblasts, while few to no gaps were present within the desmin network. 

Semiquantitative analysis (Figure 4b) revealed the percentage of myoblasts stained with different intensities. At 24 h, no significant difference in the percentage of negatively stained myoblasts were observed (*p* > 0.05). After 48 h, myoblasts exposed to 1 µM of either monensin or salinomycin had a larger percentage of negatively stained myoblasts compared to the control (*p* < 0.001). Salinomycin also had more negatively stained myoblasts compared to lasalocid (*p* < 0.5). However, at 72 h exposure, only myoblasts exposed to monensin had a significantly larger percentage of desmin-negative myoblasts compared to the control (*p* < 0.01). 

Lightly stained myoblasts had desmin filaments visible within the cytoplasm, usually located around the nucleus, but with low optical density. After 48 and 72 h exposure, both the negative control (*p* < 0.001) and lasalocid (*p* < 0.01) had significantly more lightly stained myoblasts compared to either salinomycin or monensin. 

In general, myoblasts stained and categorized as medium were similar between the control and the different ionophores over the 72 h exposure period, with exceptions between the control and monensin at 24 h (*p* < 0.05), as well as between the control and both monensin (*p* < 0.05) and salinomycin (*p* < 0.001) at 72 h. 

Finally, in case of exposure to monensin and salinomycin, a significantly (*p* < 0.05) higher percentage of myoblasts stained dark after 48 and 72 h compared to both the control and lasalocid. 

In summary, the percentage of darkly stained myoblasts remained minimal in the negative control over the 72 h exposure period, while the number of myoblasts stained with medium intensity decreased with longer exposure times. However, the number of lightly stained myoblasts increased with the exposure time. Desmin labelling of C2C12 myoblasts, exposed to either monensin or salinomycin, had a significantly larger number of myoblasts (*p* < 0.05) classified as either medium or dark. Additionally, there were more desmin-negative myoblasts after 48 and 72 h exposure to monensin and salinomycin compared to the negative control. Myoblasts exposed to 1 µM lasalocid resembled those of the negative control.

#### 2.2.2. Synemin

**Figure 5:**
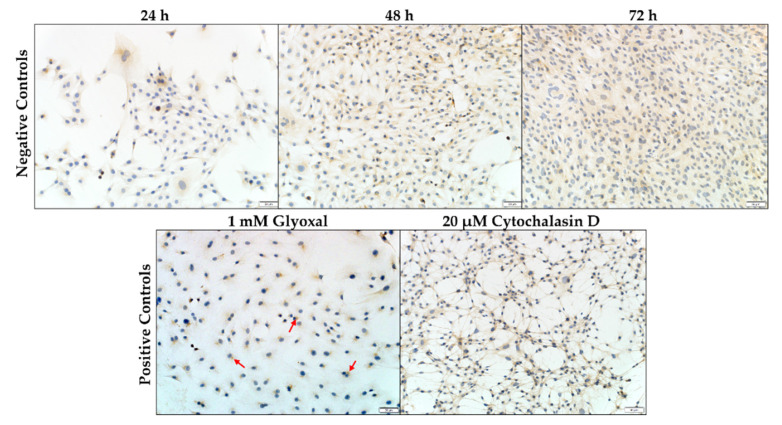
C2C12 myoblasts stained for synemin. The negative control consisted of C2C12 myoblasts incubated in DMEM medium over a 72 h period. Glyoxal and Cytochalasin D were used as positive controls. Synemin aggregation is indicated with red arrows. Scale bar = 50 µm.

As with desmin, synemin distribution within the C2C12 myoblasts were investigated using a primary antibody followed by DAB staining; however, synemin exhibited a considerably less intense brown color. The negative and solvent (not shown) controls were similar, with synemin spread diffusely across the cytoplasm (Figure 5). A few myoblasts exhibited a higher concentration of synemin in the perinuclear region. The number of C2C12 myoblasts increased over the 72 h period. Glyoxal and cytochalasin D were used as positive controls. Myoblasts exposed to 1 mM glyoxal had small, concentrated dots of synemin adjacent to the nucleus (Figure 5, red arrows). Cytochalasin D exposure resulted in the reduction in the cytoplasm volume around the nucleus with thin filamentous protrusions reaching between the myoblasts.

**Figure 6:**
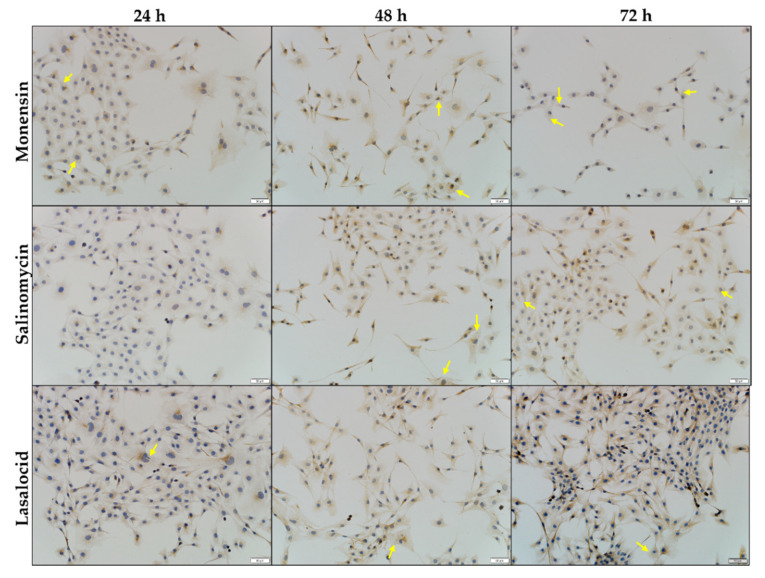
C2C12 myoblasts stained for synemin after exposure to 1 µM monensin, salinomycin and lasalocid over a period of 72 h. Yellow arrows indicate vesicles/gaps within the synemin network. Scale bar = 50 µm.

After ionophore exposure, faint gaps appeared within the synemin network of the myoblasts (Figure 6, yellow arrows). Myoblasts exposed to monensin had the greatest number of gaps, which correlated with an increase in exposure time. Lasalocid, in contrast, exhibited the lowest number of gaps. Some myoblasts were elongated, with the protrusions staining slightly darker. A few pyknotic nuclei could be seen in the samples as well. No other obvious differences could be observed between synemin staining of the negative control and ionophore-exposed myoblasts.

### 2.3. The In Vitro Effect of the Ionophores on the Microfilament and Microtubule Networks of C2C12 Myoblasts

**Figure 7:**
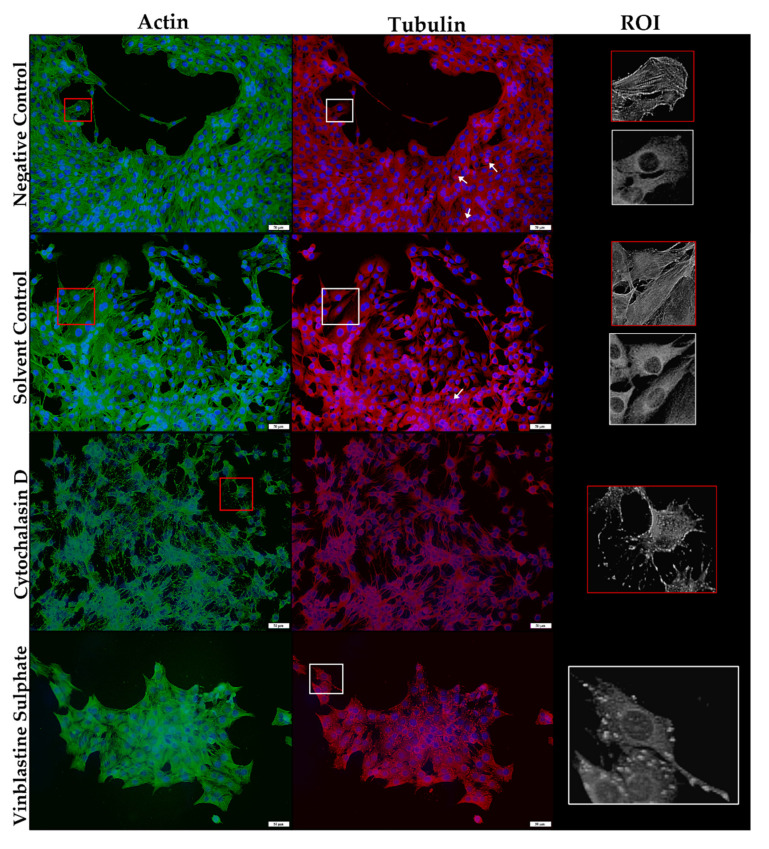
The microfilament (green) and microtubule (red) network of C2C12 myoblast controls stained using phalloidin-FITC and anti-β-tubulin-Cy3, respectively. The nuclei were counterstained with DAPI (blue). The ROI shows a close-up of selected myoblasts. White arrows indicate myoblasts undergoing mitosis. Scale bar = 50 µm.

Immunofluorescence techniques were used to investigate the effect of ionophore exposure on the microfilaments and microtubules of C2C12 myoblasts. After staining, the negative and solvent controls had a normal mesh-like arrangement of polymerized actin filaments (green) and a dense network of β-tubulin filaments (red) (Figure 7). The myoblasts generally contained one centrally located nucleus (blue) per myoblast. A few myoblasts were in the process of mitosis, as indicated by the visible mitotic spindle (white arrows). Cytochalasin D and vinblastine sulphate were used as positive controls, since they are known inhibitors of actin polymerization and microtubule formation, respectively. Cytochalasin D was added 15 min before fixation, which resulted in the disruption of the microfilament network. Typical examples of affected myoblasts are demonstrated in Figure 7, under the region of interest (ROI). Vinblastine sulphate, added an hour before fixation, resulted in a disorganized microtubular network with short, stub-like threads appearing. 

**Figure 8:**
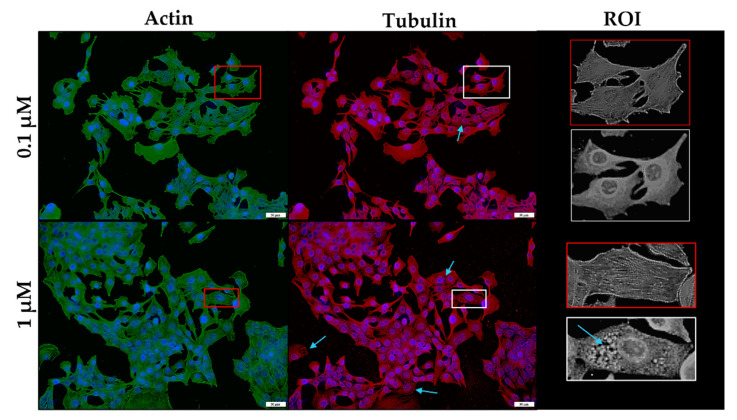
The microfilament (green) and microtubule (red) network of C2C12 myoblasts exposed to monensin for 48 h, stained using phalloidin-FITC and anti-β-tubulin-Cy3, respectively. The nuclei were counterstained with DAPI (blue). Blue arrows indicate myoblasts with gaps within the microtubule network. The ROI shows a close-up of selected myoblasts. Scale bar = 50 µm.

After exposure to 0.1 µM monensin for 48 h, the microfilament network was minimally affected (Figure 8). However, a few myoblasts showed disruption of the filamentous network after 1 µM exposure. In contrast, the microtubule network exhibited clear gaps (blue arrows) and stained darker around the nuclei of the cells compared to that of the controls. In addition to ‘rounded’ myoblasts, others appeared to be elongated.

**Figure 9:**
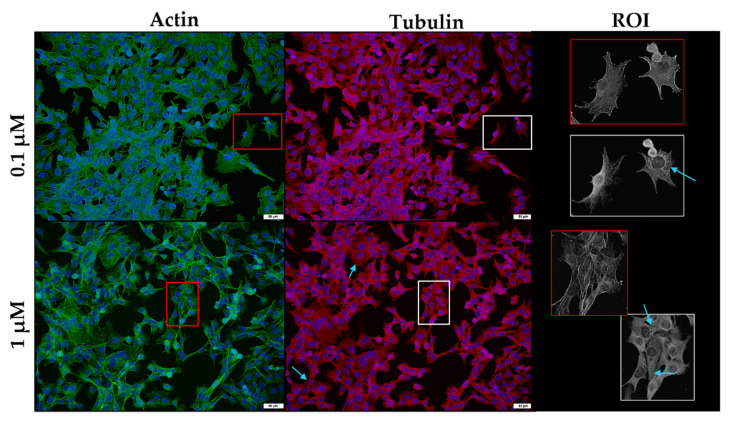
The microfilament (green) and microtubule (red) network of C2C12 myoblasts exposed to salinomycin for 48 h, stained using phalloidin-FITC and anti-β-tubulin-Cy3, respectively. The nuclei were counterstained with DAPI (blue). Blue arrows indicate myoblasts with gaps within the microtubule network. The ROI shows a close-up of selected myoblasts. Scale bar = 50 µm.

Salinomycin exposure resulted in similar, but less obvious effects on these networks within C2C12 myoblasts (Figure 9). The microtubule network contained a few gaps (blue arrows); however, the microfilament network remained mostly unaffected. As with monensin, salinomycin exposure prompted the appearance of both round and elongated myoblasts. 

**Figure 10:**
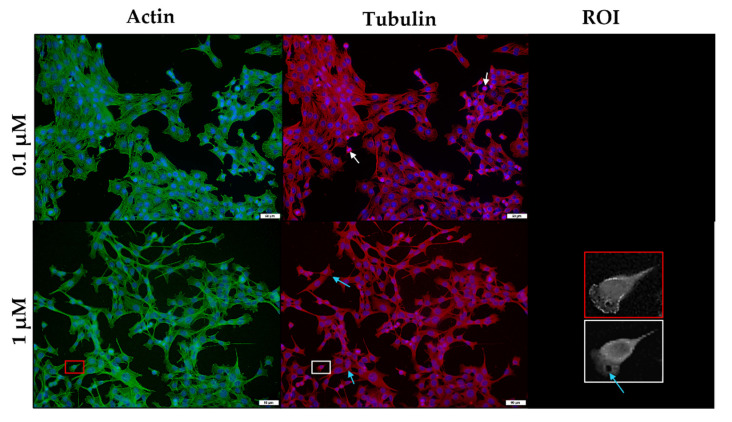
The microfilament (green) and microtubule (red) network of C2C12 myoblasts exposed to lasalocid for 48 h, stained using phalloidin-FITC and anti-β-tubulin-Cy3, respectively. The nuclei were counterstained with DAPI (blue). White arrows indicate myoblasts undergoing mitosis; blue arrows indicate myoblasts with gaps within the microtubule network. The ROI shows a close-up of selected myoblasts. Scale bar = 50 µm.

Exposure to 0.1 or 1 µM lasalocid resulted in no obvious effects in either the microfilament or microtubule network (Figure 10). A small number of myoblasts contained gaps (blue arrows) within the microtubule network; however, most remained unaffected. Noticeably, a few myoblasts were undergoing mitosis (white arrows). 

## 3. Discussion

The cytotoxicity of the carboxylic ionophores, monensin, salinomycin and lasalocid, were evaluated in C2C12 myoblasts using the MTT assay. Of the three ionophores tested, monensin had the greatest cytotoxicity, followed by salinomycin and, finally, lasalocid (Table 1). These results follow a similar trend as LD_50_ data reported in the literature [3,16,18,20,21,38]. Mice are more susceptible to salinomycin (57.4 mg/kg) compared to monensin (70–96 mg/kg); however, lasalocid (100–146 mg/kg) exhibited the lowest toxic effect, which corroborates findings of the current *in vitro* study. Pharmacokinetic aspects such as absorption, distribution, metabolism, and excretion, have a large impact on *in vivo* toxicity. After ingestion, ionophores are extensively metabolized within the liver and subsequently excreted in bile [3]. However, in the presence of drugs that interfere with the metabolic degradation of the ionophores, e.g., tiamulin [2,10], tissues are exposed to higher concentrations of ionophores and for longer periods, thus increasing their toxic effect. Comparatively, the toxicity of the ionophores, at the exposure times measured, increased considerably during the 72 h maximum exposure period, with initial EC_50_s in the micromolar range, decreasing to the low nanomolar range at longer exposure times. The *in vitro* cytotoxicity of ionophores on various cancer cell lines have previously been investigated with the EC_50_s ranging from the high micromolar range to the low nanomolar range depending on the cell line and the duration of exposure [39,40,41,42]. 

Investigation of the myoblast’s morphology using phase-contrast microscopy revealed the formation of abundant translucent vesicles following exposure to the ionophores, especially for monensin (Figure 2). In severely affected myoblasts, the entire cytoplasm was filled with these vesicles. It is surmised that the vesicles originate from the Golgi apparatus as a result of blocked cellular transport [43] or due to the disrupted osmotic balance. The Golgi apparatus is commonly located near the nuclei of the cells, possibly explaining why the vesicles usually first appear within the perinuclear region. Initially, C2C12 myoblasts are spindle-to-stellate shaped cells that can exhibit long cell-to-cell protrusions, especially noticeable in low density cultures. However, following exposure, the myoblasts ‘rounded-up’ and detached from their surroundings. Ionophores induce cytotoxicity by disruption of the ion homeostasis and resultant downstream effects [3]. Toxic ionophore concentrations can disrupt the mitochondrial membrane potential, affecting oxidative phosphorylation and increasing the production of ROS [44]. Subsequently, the lack of energy exacerbates the ion imbalance, and the ROS further damages the organelles and plasma membrane. Additionally, calcium overload, lipid peroxidation and altered cellular pH also contributes to cellular toxicity [3]. 

In this study, monensin and salinomycin had a greater cytotoxic effect on the myoblasts compared to lasalocid, which agrees with the cytotoxic effects observed within the cytoskeletal network after exposure to these ionophores. For example, some of the C2C12 myoblasts exposed to lasalocid still continued to undergo mitosis, indicating that proliferation was still ongoing (Figure 10). As the EC_50_s obtained for lasalocid was above 1 µM, it is not unexpected that a few myoblasts were actively proliferating.

After monensin and salinomycin exposure, the desmin intermediate filament aggregated perinuclearly (Figure 4). Desmin is a critical component in the myofiber network, and various myopathies are associated with its disruption and aggregation [27]. Additionally, desmin plays a role in determining the intracellular location of mitochondria and regulating their respiratory function [29,30]. Therefore, the disruption and loss of desmin filaments could potentially exacerbate the energy deficiency caused by ionophore exposure. 

The integrity of the microtubule network was slightly affected as well. Gaps were visible within the microtubule network and the microtubules were concentrated around the nuclei of the myoblasts (Figure 8, Figure 9 and Figure 10). The gaps correspond to vesicles generated after ionophore exposure (Figure 2). As previously mentioned, ionophore exposure *in vitro* disrupts cellular transport and increases the number of vesicles found within the cytoplasm [43]. Various functions such as the transportation of cellular “cargo” and mitosis require a functioning microtubular network. Increased cytoplasmic calcium could also potentially disrupt these functions as it results in decreased microtubule formation [45,46]. For example, both axonal transport and the quantity of microtubules were decreased in frog sciatic nerves after exposure to lasalocid, which increased intracellular calcium concentrations [47]. 

The synemin and microfilament networks were not greatly affected by ionophore exposure, except for the slight gaps within both networks. 

The cytoskeleton performs an important role in the structural integrity and functioning of muscle. Ultrastructural studies of skeletal muscles of animals affected following ionophore exposure reported myofibrillar degeneration and necrosis, with disrupted contractile apparatus [22,23]. The disruption in the desmin intermediate filament network observed in the current study could thus contribute to the myofibrillar degeneration previously reported, as desmin is primarily associated with the Z-disk of striated muscle [25,26,27,28]. 

Desmin is also a substrate for various calcium-activated proteases, such as calpains, which could potentially be responsible for the degradation of desmin filaments. Salinomycin reportedly activates calpain in murine dorsal root ganglion neurons [48]. In contrast, a study using leupeptin, a protease inhibitor, failed in protecting frog skeletal muscles from damage after exposure to the ionophore, calcimycin, *in vitro*, implying that myofibrillar damage might be due to another mechanism other than calcium activated proteases [49]. Future studies could focus on whether ionophores activate calpain within myoblasts and muscle tissues. 

## 4. Conclusions

Of the three carboxylic ionophores investigates, monensin was the most cytotoxic *in vitro*, followed by salinomycin and lasalocid. The main cytoskeletal element of C2C12 myoblasts affected by ionophore exposure was the desmin intermediate filament network. The microtubule network was also affected, but to a lesser extent. 

## 5. Materials and Methods

### 5.1. Cell Culture

The mouse skeletal muscle cell line, C2C12, obtained from the American Type Culture Collection (ATCC^®^ CRL-1772^TM^) was used for this study. The myoblasts were cultured in Dulbecco’s Modified Eagle’s Medium (DMEM) (PAN Biotech, Aidenbach, Germany) supplemented with 100 U penicillin/mL and 100 U streptomycin/mL (Lonza, Verviers, Belgium) and 10% fetal calf serum (Gibco, Origin Brazil, Grand Island, NY, USA). The myoblasts were incubated in a humidified atmosphere with 5% CO_2_, at 37 °C and periodically sub-cultured to prevent the culture from reaching confluency.

### 5.2. Exposure to the Ionophores

C2C12 myoblasts were seeded into 96-well plates at a concentration of 10 000 myoblasts/mL and incubated for 24 h to allow the myoblasts time to attach and reach the log-phase of growth prior to commencing with exposure. 

Monensin sodium (MW: 692.85 g/mol), salinomycin sodium (MW: 772.98 g/mol) and lasalocid A sodium (MW: 612.77 g/mol) were obtained from Dr Ehrenstorfer^®^ (Augsburg, Germany). The purity of the ionophores, as indicated on the certificate of analysis, were 98.4, 78.8 and 96.8%, respectively.

As the ionophores were easily solubilized in MeOH, stock solutions were prepared by dissolving the ionophores in MeOH up to a concentration of 40 mM. The solutions were then further diluted with DMEM media, with a final solvent concentration of 0.1% MeOH. The cytotoxic effect of the ionophores were determined after 24, 48 and 72 h.

### 5.3. Cell viability Assay

Cell viability was measured using a modified MTT assay first described by Mosmann et al. (1983) [50]. Briefly, post exposure, the 96-well plates were washed with phosphate-buffered saline (PBS) (Sigma-Aldrich, St. Louis, MO, USA), followed by the addition of 200 µL complete DMEM medium and 20 µL 5 mg/mL MTT (in PBS) (Sigma-Aldrich, St. Louis, MO, USA). Incubation was continued for 2 h in the dark, in a humidified atmosphere with 5% CO_2_, at 37 °C, before the medium was decanted and 100 µL DMSO added to each well. The absorbance and background absorbance were read at 570 nm and 630 nm, respectively, using a Synergy HT BioTek microplate reader (BIO-TEK Instruments, Winooski, VT, USA). Viability was expressed as a percentage of the solvent control after subtracting the media control. Additionally, doxorubicin was used as a positive control for the MTT assay (not shown).

### 5.4. Immunocytochemical Detection and Analysis of Desmin and Synemin Intermediate Filaments

C2C12 myoblasts were cultured in DMEM supplemented with 10% FBS, 100 U penicillin/mL and 100 U streptomycin/mL. Myoblasts were seeded into an 8-well chamber slide (10 000 cells/mL) and allowed 24 h to attach to the surface, before being exposed to 1 µM monensin, salinomycin and lasalocid for 24, 48 and 72 h, respectively. Due to the varying cytotoxicity between the different ionophore and exposure times, it was decided to use 1 µM as it falls slightly below the EC_50_s of lasalocid, but above those of monensin and salinomycin after 48 and 72 h exposure. The controls used during this study included a negative control (complete DMEM medium), a solvent control (0.1% MeOH) and two positive controls (1 mM glyoxal added on the day of exposure, as well as 20 µM cytochalasin D added 30 min before fixation). 

After exposure, immunocytochemistry was performed using an indirect immunoperoxidase technique as previously described by Botha et al. (2019) [51]. The slides were air-dried and fixed in 4% neutral buffered formalin for at least 30 min, followed by rinsing the slides using 70% alcohol for an additional 10 min. To suppress any endogenous peroxidase activity, each slide was incubated in 3% hydrogen peroxide (in MeOH) for 15 min. Microwave heat-induced epitope retrieval (HIER) followed, using Tris-EDTA (pH 9) and citrate (pH 6) buffer solutions, for desmin and synemin staining, respectively. To facilitate non-specific immunoglobulin binding, slides were incubated with normal horse serum (1:10 in PBS, pH 7.6) containing 0.1% bovine serum albumin (BSA) for 20 min in a humidified chamber at room temperature. The myoblasts were incubated with either the mouse monoclonal desmin (D33) antibody (1:200) (DakoCytomation, Glostrup, Denmark) or the goat polyclonal synemin (S-16) antibody (1:100) (Santa Cruz Biotechnology, Inc. Santa Cruz, CA, USA) for 1 h. Following this, slides incubated with the synemin polyclonal antibody were incubated with a rabbit anti-goat antibody (Dako, Glostrup, Denmark), diluted 1:500 in PBS containing 0.1% BSA, for 30 min in a humidified chamber at room temperature. All slides were incubated with the Dako REAL Envision Rabbit/Mouse polymer-based detection system (Dako, Glostrup, Denmark) performed according to the manufacturer’s instructions. The slides were incubated with DAB chromogen for 1–2 min, rinsed with distilled water, counter stained with hematoxylin for 15 sec and washed again with distilled water. Finally, slides were dehydrated with 70%, 96% and 100% alcohol and mounted using Entellan (Merck Millipore, Darmstadt, Germany) and coverslips for examination under a microscope. Each slide was viewed and photographed using the BX63 Olympus microscope (Tokyo, Japan). Six (692 × 521 µm) images were taken and used for further analysis. Semiquantitative analysis on the staining intensity of desmin was performed using QuPath (Version 0.3.0, open-source software) [52] positive cell detection function (Figure 4b). The staining intensity was categorized based on different threshold values of the DAB optical density mean. The total number of nuclei per image was counted and classified as negative (<0.2), light (0.2–0.5), medium (0.5–1.0) or dark (>1.0) depending on the optical density around the nucleus. Myoblasts categorized as negative had no desmin surrounding the nucleus. Each image was then manually scanned for errors and corrected where necessary. The percentages were calculated as a function of the total number of myoblasts counted, then averaged over the six images.

### 5.5. Immunofluorescent Detection of Actin and β-Tubulin

Immunofluorescent staining was performed as previously described by Botha et al. (2019) [51]. The C2C12 myoblasts were seeded onto sterile 10 mm-diameter coverslips placed in a 24-well plate and given 24 h to adhere to the coverslips. The myoblasts were exposed to 0.1 and 1 µM monensin, salinomycin and lasalocid, respectively, for 48 h. Cytochalasin D (Sigma-Aldrich, St. Louis, MO, USA) and vinblastine sulphate (Sigma-Aldrich, St. Louis, MO, USA) were used as positive controls for microfilament and microtubule network disruption, respectively. The myoblasts were exposed to 12 µM cytochalasin D for 15 min and 5 µM vinblastine sulphate for an hour at 37 °C, just prior to fixation. Exposure was followed by a washing step, gently shaking the myoblasts in PBS for 5 min, before fixing the myoblasts with 100% ice-cold acetone (Merck) at −20 °C for 10 min. The acetone was removed, and the 5 min wash step was repeated twice. Actin was stained by the addition of 1 µg/mL phalloidin-fluorescein isothiocyanate (FITC) (Sigma-Aldrich, St. Louis, MO, USA) prepared in PBS and incubated for 30 min at 37 °C. Before staining for β-tubulin, the myoblasts were first incubated with 1% BSA (Biowest, Nuaillé, France) in PBS to block all nonspecific binding cites, followed by incubation with 1 µg/mL (1:100 in PBS) monoclonal anti-β-tubulin-Cy3 (Sigma-Aldrich, St. Louis, MO, USA) for 1 h at 37 °C. After washing three times with PBS to remove the excess stain, the nuclei were counterstained with 1.3 µg/mL DAPI (in ddH_2_O) for 15 min at 37 °C. The washing step was then again repeated 3 times. Finally, the coverslips were mounted on a microscope slide with Prolong^TM^ Gold antifade reagent (Molecular Probes, Eugene, OR, USA) and sealed to prevent drying of the sample. The samples were viewed with an Olympus BX63 Fluorescent microscope (Tokyo, Japan). The fluorescent images were merged using Fiji–ImageJ (open-source software) [53]. The images were deconvoluted using the Diffraction PSF 3D and Iterative Deconvolve 3D plugins [54].

### 5.6. Statistical Analysis

All EC_50_s were calculated using the non-computational method first described by Alexander et al. (1999) [55]. Further statistical analysis was performed using GraphPad Prism version 9.0 (GraphPad Software, San Diego, CA, USA). Normality was determined using the Shapiro–Wilk normality test. Significant differences were determined using analysis of variance (ANOVA), followed by an ad hoc Tukey’s test, to determine differences between individual samples from the cytotoxicity assays as well as the semi-quantitative desmin analysis.

## Figures and Tables

**Table 1:** The EC_50_s (µM) of monensin, salinomycin and lasalocid on C2C12 myoblasts reported as the mean ± the standard error of the mean.

		Monensin	Salinomycin	Lasalocid
C2C12 cells	24 h	† (*n* = 5)	4.04 ± 1.01 (*n* = 5) ^a^	8.68 ± 5.38 (*n* = 4) ^a^
	48 h	0.04 ± 0.01 (*n* = 5) ^c^	0.45 ± 0.13 (*n* = 5) ^b,d^	1.38 ± 0.54 (*n* = 4) ^b,d^
	72 h	0.02 ± 0.01 (*n* = 5) ^c^	0.26 ± 0.06 (*n* = 5) ^b,d^	1.46 ± 0.59 (*n* = 4) ^b,d^

† The EC_50_ falls between 0.15 and 10 µM. *n* = biological repeats. Significant differences between the EC_50_s of the ionophores as well as between the EC_50_s at different exposure periods are indicated using ^a & b^ (vertical columns) and ^c & d^ (horizontal columns), respectively.

## Data Availability

Not applicable.
